# Amaranth’s Growth and Physiological Responses to Salt Stress and the Functional Analysis of *AtrTCP1* Gene

**DOI:** 10.3390/ijms25105437

**Published:** 2024-05-16

**Authors:** Shengcai Liu, Zixian An, Zhongxiong Lai

**Affiliations:** 1 Institute of Horticultural Biotechnology, Fujian Agriculture and Forestry University, Fuzhou 350002, China; annzixian@163.com; 2 Key Laboratory of Ministry of Education for Genetics, Breeding and Multiple Utilization of Crops, Fujian Agriculture and Forestry University, Fuzhou 350002, China

**Keywords:** amaranth, salt stress, growth, physiology, *TCP1*, molecular function

## Abstract

Amaranth species are C4 plants that are rich in betalains, and they are tolerant to salinity stress. A small family of plant-specific TCP transcription factors are involved in the response to salt stress. However, it has not been investigated whether amaranth *TCP1* is involved in salt stress. We elucidated that the growth and physiology of amaranth were affected by salt concentrations of 50–200 mmol·L^−1^ NaCl. The data showed that shoot and root growth was inhibited at 200 mmol·L^−1^, while it was promoted at 50 mmol·L^−1^. Meanwhile, the plants also showed physiological responses, which indicated salt-induced injuries and adaptation to the salt stress. Moreover, *AtrTCP1* promoted *Arabidopsis* seed germination. The germination rate of wild-type (WT) and *35S::AtrTCP1-GUS Arabidopsis* seeds reached around 92% by the seventh day and 94.5% by the second day under normal conditions, respectively. With 150 mmol·L^−1^ NaCl treatment, the germination rate of the WT and *35S::AtrTCP1-GUS* plant seeds was 27.0% by the seventh day and 93.0% by the fourth day, respectively. Under salt stress, the transformed *35S::AtrTCP1* plants bloomed when they grew 21.8 leaves after 16.2 days of treatment, which was earlier than the WT plants. The transformed *Arabidopsis* plants flowered early to resist salt stress. These results reveal amaranth’s growth and physiological responses to salt stress, and provide valuable information on the *AtrTCP1* gene.

## 1. Introduction

Salinity is an important environmental stress factor that has adverse effects on plant growth and yield [1,2]. Under salt stress, plants undergo morphological, physiological, and metabolic changes [3,4,5,6,7,8], including stomatal closure, a reduction in photosynthetic rate, the inhibition of plant growth and development, and a significant loss of yield and quality. Chlorophyll is the main pigment for photosynthesis in higher plants, and it is directly related to leaf photosynthesis and plant yield potential. Salt stress reduces the leaf chlorophyll content [9]. Chlorophyll fluorescence is closely related to leaf photosynthesis [10], which reflects the effects of environmental factors on plants [11,12,13,14].

The plant root is the main organ for absorbing nutrients and water. Under salt stress, the roots are directly damaged. Salt stress can inhibit the cell cycle, cell elongation, and root meristem activity, thereby inhibiting root growth [15,16], including root length, the number of lateral roots, and root surface area. Furthermore, salt stress affects nutrient and water uptake by the roots [17,18].

Salt stress can activate reactive oxygen species (ROS) mechanisms in plants [19]. Excessive ROS are produced in plants under (a)biotic stress, which causes oxidative stress and abnormal growth. To protect them from deleterious oxidative stress, plants have evolved defense mechanisms to protect themselves against free-radical-induced damage by using a wide array of non-enzymatic and enzymatic systems [19,20,21,22,23]. Salt stress can increase the activity levels of antioxidant enzymes, thereby reducing peroxidation in cells and improving the salt tolerance of plants [22,24,25,26,27]. Additionally, salt stress changes the content and composition of secondary metabolites in plants, such as betalains [28], carotenoids [29], and flavonoids [30,31]. These secondary metabolites are involved in osmotic regulation in plants, thereby enhancing the salt tolerance of plants. Betalains are important secondary metabolites. They not only play a role in coloration, but also act as osmoregulatory substances in plants to adapt to salt stress. They directly participate in osmoregulation to maintain normal cell metabolism under salt-stress conditions [32]. Betalain production in lalshak was increased under saline stress [33].

The small family of plant-specific TCP transcription factors includes TEOSINTE BRANCHED1 (TB1) in *Zea mays*, CYCOLOIDEA (CYC) in *Antirrhinum majus*, and PROLIFERATING CELL FACTORS 1 and 2 (PCF1 and PCF2) in *Oryza sativa*, which perform comprehensive functions in plant growth and development, such as branching [34,35,36], leaf morphogenesis [37,38,39], flower development [40,41], and hormone pathway generation [42]. Additionally, TCP genes are also involved in the response to exogenous factors such as salt stress and other abiotic stresses [43,44,45,46,47,48,49,50]. The PCF gene, which is found in *Oryza sativa*, activates the expression of the *NHX* gene, thereby improving the plants’ salt tolerance [51].

Amaranth species are C4 plants that are rich in betalains, and they are tolerant to adverse environmental conditions, including droughts, saline soil, and high and low temperatures [52]. It is particularly important to uncover the key genes of salt tolerance in amaranth. Our previous study showed that the *AtrTCP*1 gene is highly expressed under salt stress using qRT-PCR analysis [53], which implies it responds to salt stress. However, the function of the *AtrTCP*1 gene is not clear. We studied the effects of salt stress on the growth and physiological characteristics of amaranth, analyzed the molecular function of *AtrTCP1*, and clarified the effects of salt stress on the growth and nutrients of amaranth and the role of *TCP1* under salt stress. This work would help us to study the profound functions of the AtrTCPs in the future.

## 2. Results

### 2.1. Salt Stress Suppressed Amaranth Growth

**Figure 1:**
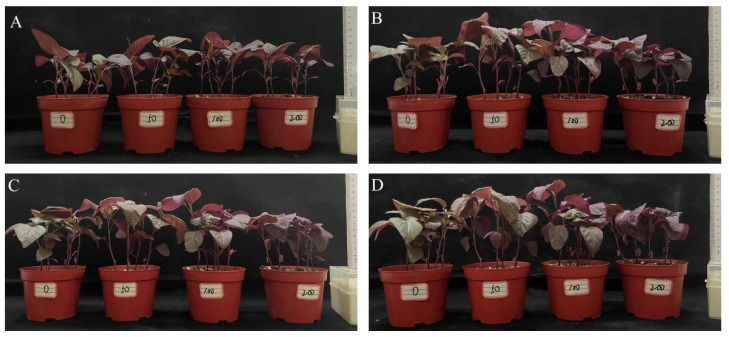
Effects of salt stress on phenotype of amaranth plants. Note: (**A**) represents amaranth before treatment; (**B**) represents amaranth plant after 3 days; (**C**) represents amaranth plant after 6 days; (**D**) represents amaranth plant after 9 days.

We selected amaranth plants at similar growth stages for the salt stress treatment (Figure 1A). Salt stress had an effect on their height (Figure 1B–D, Table 1). Three days after the 200 mmol·L^−1^ NaCl treatment, the amaranth plants were significantly shorter than those under the other treatments (Figure 1B). At 6 d and 9 d, the amaranth plants treated with 50 mmol·L^−1^ or 100 mmol·L^−1^ NaCl were significantly taller than those given the other treatments, and they were the shortest under the 200 mmol·L^−1^ NaCl treatment (Figure 1C,D). Salt stress reduced the height of the amaranth plants.

### 2.2. Salt Stress Inhibited Amaranth Root Growth

**Figure 2:**
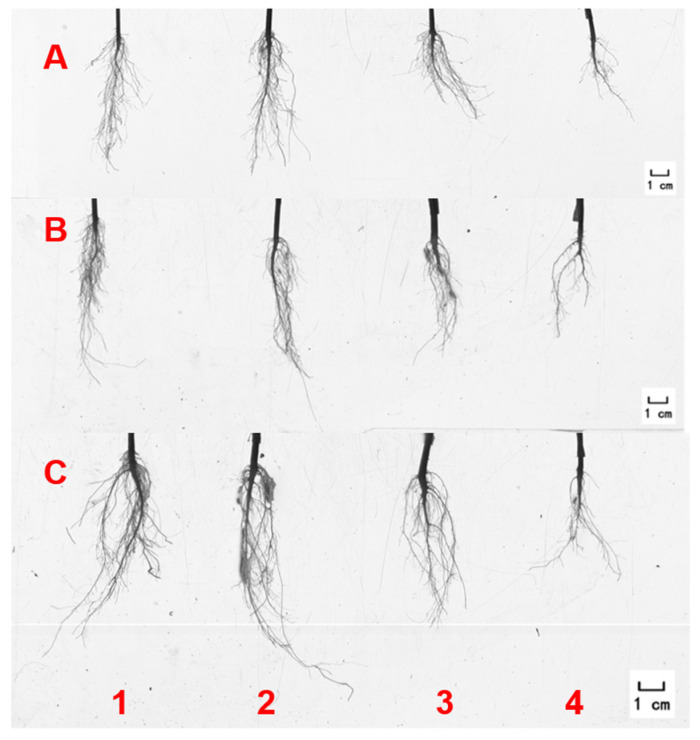

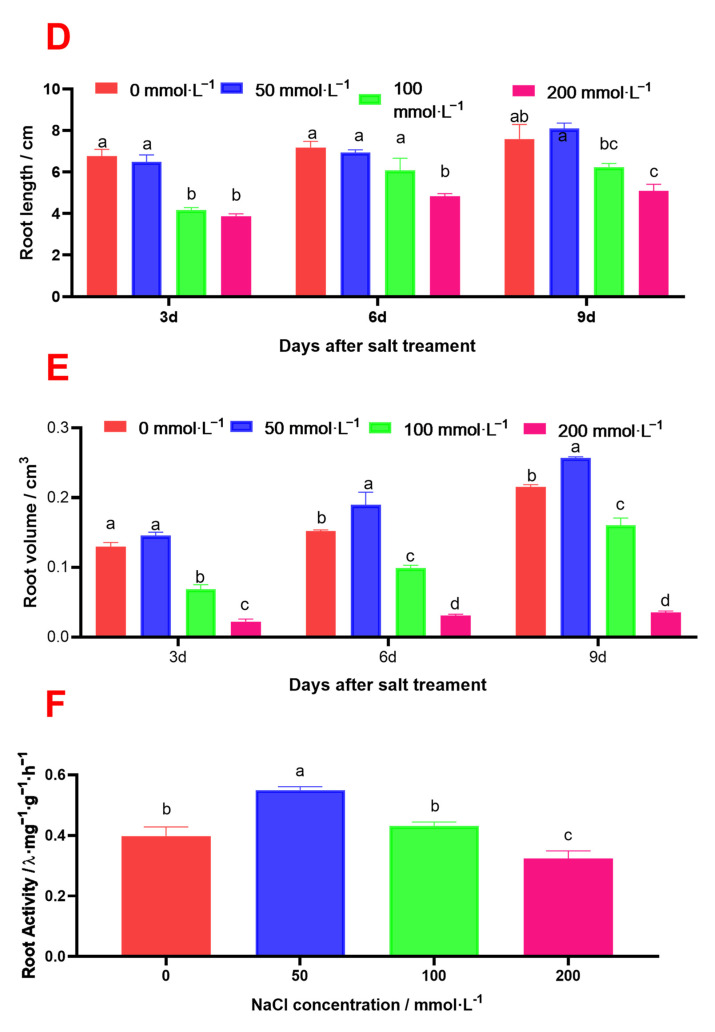
Amaranth roots under salt stress. Note: (**A**–**C**) represents roots 3 days, 6 days, and 9 days after salt stress treatment. The numbers 1, 2, 3, and 4 represent 0 mmol·L^−1^, 50 mmol·L^−1^, 100 mmol·L^−1^, and 200 mmol·L^−1^ NaCl, respectively. (**D**) represents root length. (**E**) represents root volume. (**F**) represents root activity. Data are presented as mean ± standard error. Different lowercase letters represent significant differences at the 0.05 level.

There were significant differences in the roots of the amaranth plants treated with NaCl solution. The roots after the 50 mmol·L^−1^ NaCl treatment were significantly longer, stronger, and more fibrous than those of the other treatments. However, under the treatment of 200 mmol·L^−1^ NaCl, the amaranth had the shortest roots and the fewest fibrous roots (Figure 2A–D). Similar results were obtained for the root volume and activity under salt stress. The root volume of the amaranth plants with the 50 mmol·L^−1^ NaCl treatment was five to six times larger than that of the amaranth plants treated with 200 mmol·L^−1^ NaCl (Figure 2E). Root vitality was also significantly increased at 50 mmol·L^−1^ NaCl (Figure 2F).

### 2.3. Effects of Salt Stress on the Contents of Betacyanin, Betaxanthin, Flavonoid, and Chlorophyll in Amaranth

**Figure 3:**
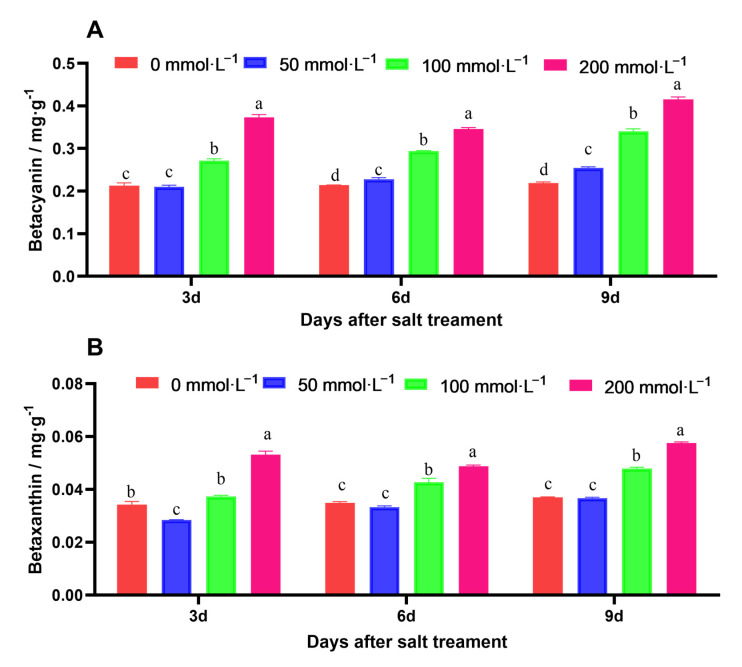
Effects of salt stress on the content of betalains. (**A**) represents betacyanin. (**B**) represents betaxanthin. Data are presented as mean ± standard error. Different lowercase letters represent significant differences at the 0.05 level.

Salt stress increased the betacyanin and betaxanthin contents in amaranth. The contents of betacyanin and betaxanthin were significantly higher than those of the control and other concentrations. Compared to the control plants, the 200 mmol·L^−1^ NaCl treatment increased the contents of betacyanin and betaxanthin by about 1.5 times (Figure 3).

**Figure 4:**
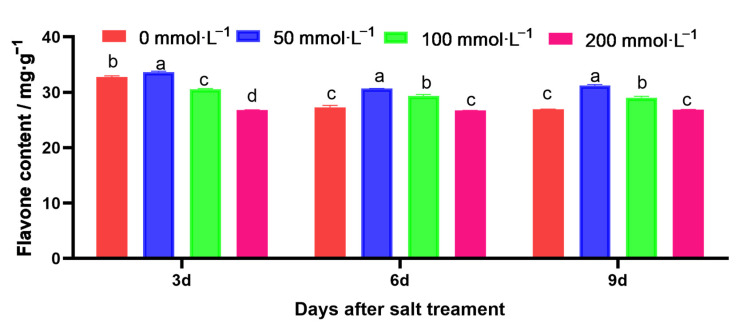
Effects of salt stress on the content of flavonoids. Data are presented as mean ± standard error. Different lowercase letters represent significant differences at the 0.05 level.

However, the flavonoid content showed a trend of ‘up/down’ with an increase in the salt solution concentration; it was the highest and lowest under the treatments with 50 mmol·L^−1^ NaCl and 200 mmol·L^−1^ NaCl, respectively (Figure 4).

**Figure 5:**
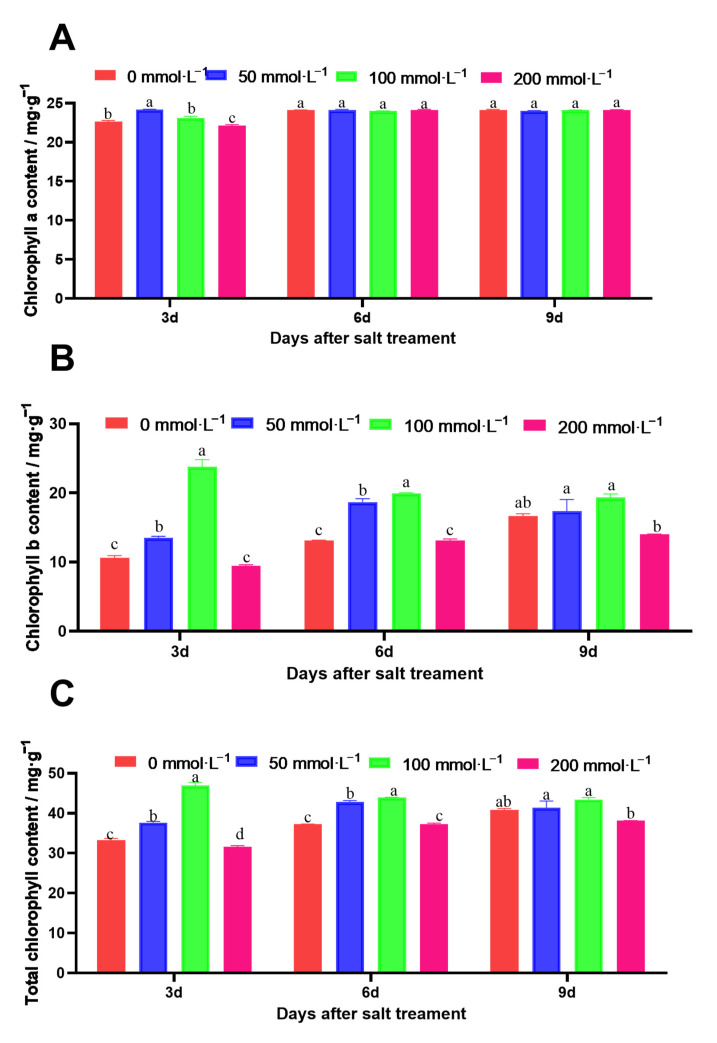
Effects of salt stress on the chlorophyll contents in amaranth. (**A**) represents chla. (**B**) represents chlb. (**C**) represents total chlorophyll. Data are presented as mean ± standard error. Different lowercase letters represent significant differences at the 0.05 level.

There was no significant difference in the content of chla in the amaranth leaves under the different salt stress concentrations. However, the content of chlb in the amaranth leaves was significantly inhibited after 3 days of the 200 mmol·L^−1^ NaCl solution treatment, which resulted in a reduction in the total chlorophyll content (Figure 5). 

### 2.4. Effect of Salt Stress on the Leaf Antioxidant Enzyme Activity and MDA Content

**Figure 6:**
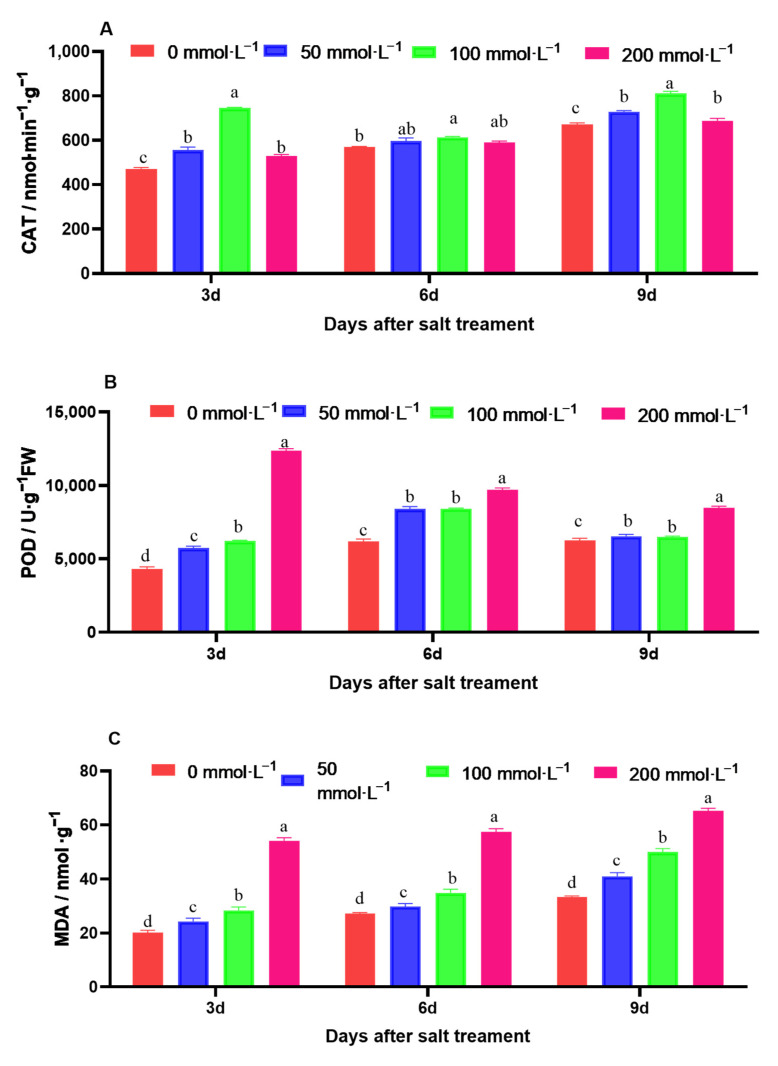
Effect of salt stress on enzyme activity and MDA content in amaranth. Note: (**A**) represents catalase activity; (**B**) represents peroxidase (POD) activity; (**C**) represents malondialdehyde (MDA) content. Data are presented as mean ± standard error. Different lowercase letters represent significant differences at the 0.05 level.

The antioxidant enzyme activity was influenced by salt stress. In comparison with that in the control, the antioxidant enzymatic activity of CAT and POD was significantly increased under salt stress, and it gradually increased over time (Figure 6A,B). The maximum increase in CAT activity in amaranth was observed for the 100 mmol·L^−1^ NaCl salt solution. The maximum increase in POD in amaranth was observed for the 200 mmol·L^−1^ NaCl salt solution. The MDA content in amaranth continuously increased during the salt stress test, whereas that of the control did not. The maximum increase in MDA in amaranth was observed for the 200 mmol·L^−1^ NaCl salt solution (Figure 6C).

### 2.5. Functional Analysis of AtrTCP1 Gene of Amaranth Transformed into Arabidopsis

**Figure 7:**
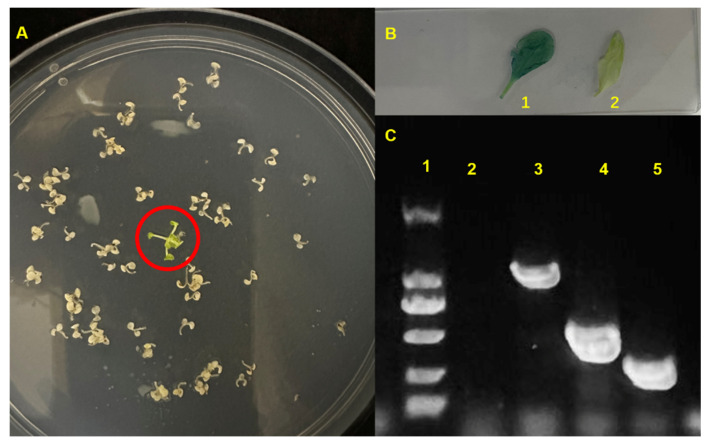
Screening of *35S::AtrTCP1* plants. (**A**) represents the positive plants screened on the selected medium. (**B**) represents Gus staining, 1 denotes *35S::AtrTCP1*, and 2 indicates the WT seedlings. (**C**) represents the dentification of positive *Arabidopsis* plants using PCR; the number 1 represents DL2000, 2 denotes the WT seedlings, 3 indicates the *AtrTCP1* gene, 4 marks the *Hyg* gene, and 5 signifies the *GUS* gene.

We obtained the cDNA sequence of *AtrTCP1* using RT-PCR; it is 1095 bp long, containing 364 aa. The sequence was submitted to NCBI (GenBank Number: PP681416). Meanwhile, we constructed an overexpression recombinant plasmid vector and transmitted it to *Agrobacterium tumefaciens*. Then, *35S:AtrTCP1-GUS* was transformed into *Arabidopsis* by using the floral dip method. The positive plants were identified through the resistance screening (Figure 7A), GUS staining (Figure 7B), and PCR amplification of *AtrTCP1*, *GUS,* and *Hyg* genes (Figure 7C). The results showed that only transgenic *Arabidopsis* could root on the selected medium, and the leaves were stained blue using GUS staining. Furthermore, the corresponding bands were also determined using PCR amplification.

**Figure 8:**
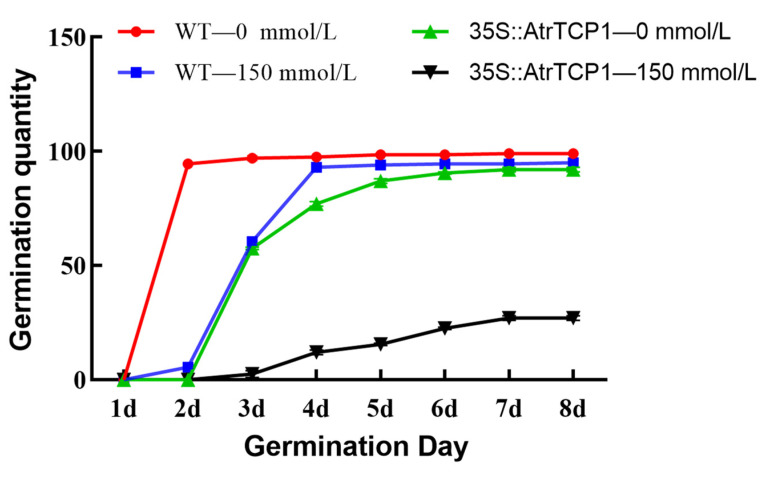
Effects of salt stress on seed germination of wild-type and transgenic plants.

To explore the function of *AtrTCP1* in response to salt stress, we compared the germination rates of transgenic AtrTCP1 and wild-type (WT) plant seeds under this condition. The germination rates and timings of the *35S::AtrTCP1*-GUS plants were different from those of the WT plants. The germination rate of the WT and *35S::AtrTCP1*-GUS plant seeds reached around 92% on the seventh day and 94.5% on the second day under normal conditions, respectively (Figure 8). Under the treatment of 150 mmol·L^−1^ NaCl, the germination rate of the WT and *35S::AtrTCP1*-GUS plant seeds was 27.0% on the seventh day and 93.0% on the fourth day, respectively (Figure 8). 

**Figure 9:**
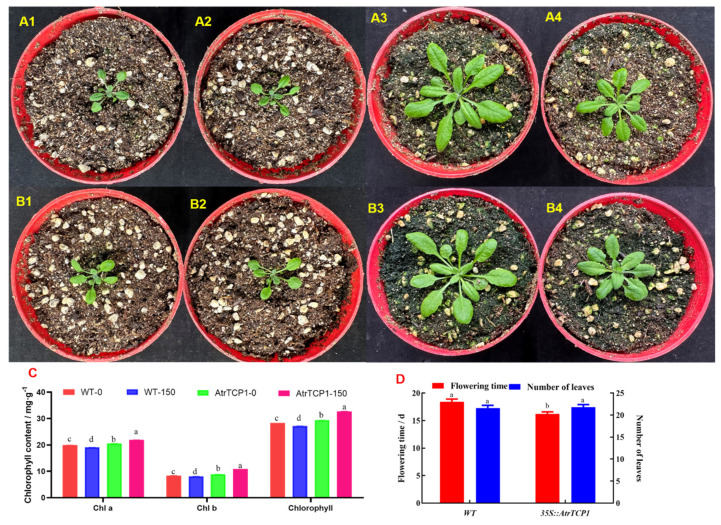
Effects of salt stress on *Arabidopsis* plants. Note: (**A1**,**A2**) represent wild-type *Arabidopsis* plants before salt treatment. (**A3**,**A4**) represent wild-type *Arabidopsis* plants after 0 mM and 150 mM NaCl treatment for 7 days, respectively. (**B1**,**B2**) represent *35S::AtrTCP11 Arabidopsis* plants before salt treatment. (**B3**,**B4**) represent *35S::AtrTCP1 Arabidopsis* after 0 mM and 150 mM NaCl treatment for 7 days, respectively. (**C**) represents chlorophyll content. (**D**) represents flowering time and leaf number. Data are presented as mean ± standard error. Different lowercase letters represent significant differences at the 0.05 level.

Subsequently, we compared the plant growth of the transgenic *35S::AtrTCP1* and wild-type (WT) seeds (shown in Figure 9). The transgenic *35S::AtrTCP1* and WT plants were shorter in the salt stress test than they were under normal conditions, and they had fewer leaves (Figure 9A,B). Furthermore, the contents of chlorophyll a, chlorophyll b, and total chlorophyll in the salt-stress-induced plants were significantly lower than those in the non-salt-stress-induced plants. However, the contents of chlorophyll a, chlorophyll b, and total chlorophyll in the transformed *35S::AtrTCP1* plants were significantly higher than those not under salt stress. The chlorophyll content of the transformed plants was higher than that of the wild-type ones under salt stress and not under salt stress (Figure 9C). 

The *AtrTCP1* gene might not influence leaf growth or flowering time when plants are not under salt stress because the transformed *35S::AtrTCP1* plants bloomed and the WT ones grew about 16 leaves after 13 days of treatment. Interestingly, under salt stress, the transformed *35S::AtrTCP1* plants bloomed when they grew 21.8 leaves after 16.2 days treatment. However, the wild type flowered when they grew 21.6 leaves after 18.4 days treatment. This implies that salt stress delayed the flowering time in *Arabidopsis*, but *35S::AtrTCP1* promoted blooming (Figure 9D).

**Figure 10:**
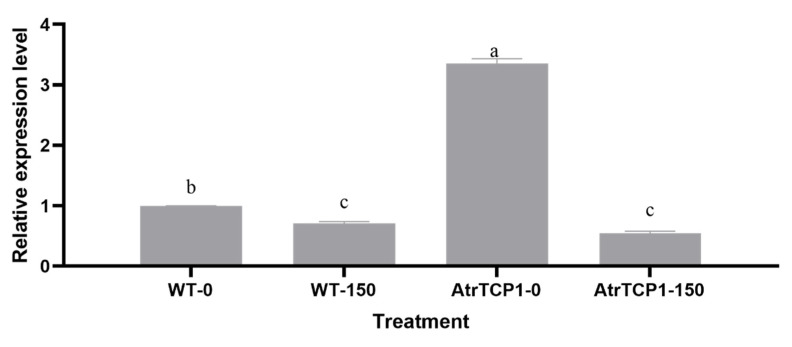
Effects of salt stress on *Arabidopsis* plants. WT-0 and WT-150 represent wild-type *Arabidopsis* plants without salt stress treatment and with 150 mM NaCl treatment, respectively. AtrTCP1-0 and AtrTCP1-150 represent *35S::AtrTCP1 Arabidopsis* plants under without salt stress treatment and with 150 mM NaCl treatment, respectively. Data are presented as mean ± standard error. Different lowercase letters represent significant differences at the 0.05 level.

Because the *AtrTCP1* gene in amaranth does not exist in *Arabidopsis*, we designed a pair of primer for detecting the *AtrTCP1* expressional level according to the *AtTCP20* and *AtrTCP1* gene sequences, which could detect both the *AtrTCP1* and *AtTCP20* expressional level. The expression of the *AtrTCP1* genes in the transformed *Arabidopsis* leaves was significantly higher than the WT for those not under salt stress. Under salt stress, the expression of the *AtrTCP1* genes in the WT and transformed *35S::AtrTCP1* plants was lower than that of those not under salt stress (Figure 10). Perhaps under salt stress, *AtrTCP1* was down-regulated in *Arabidopsis*, but it promoted blooming earlier to reduce the duration of salt stress. We speculated that the transformed plants flowered early to resist salt stress.

## 3. Discussion

Photosynthesis is involved in energy metabolism during plant growth and development. Chlorophyll is the key indicator of a plant’s photosynthetic capacity [54]. Stress can inhibit chlorophyll biosynthesis and accelerate chlorophyll degradation, thereby affecting the plants’ photosynthesis [55,56]. We found that the increase in chlorophyll content increased with the 50 mmol·L^−1^ NaCl treatment, while the chlorophyll content decreased under the treatment with 200 mmol·L^−1^ NaCl. This result was consistent with *Populus talassica* × *Populus euphratica* [57]. Under a high salt concentration, salt interferes with the photosynthetic process of plants and photosynthetic enzyme activities, resulting in a reduction in photosynthetic activity and the destruction of the chloroplast structure, which, in turn, affects the synthesis of chlorophylls [58]. Stress can reduce the chlorophyll content and promote chlorophyll degradation, which further influences photosynthesis. However, antioxidant enzymes inhibit chlorophyll degradation under stress [59]. 

Enzyme activity is an important indicator of the damage of plants under stress, which directly reflects the physiological and biochemical changes in plants. Toxic elements (Zn) reduce the contents of chlorophyll and proline and affect the root system in *Cannabis sativa* [60]. Under salt stress, ROS accumulate excessively, which could damage the plants. Antioxidants, such as peroxidase (POD), catalase (CAT), and superoxide dismutase (SOD), play an important role in eliminating ROS and protecting the plant from oxidative stress [61,62,63]. We verified that the POD activity increased with an increase in salt concentration, which indicates that salt stress induces oxidative stress in plants. MDA is an important indicator of oxidative damage in plants caused by stress. The higher the salt concentration is, the more serious the damage to the membrane is, and the MDA content increases significantly [64]. We verified that the MDA content increased with an increase in salt concentration, which perhaps aggravated the oxidative degradation of the membrane.

Salt stress changes the content and composition of secondary metabolites in plants, such as betalains [28], carotenoids [29], and flavonoids [30,31]. These secondary metabolites are involved in osmotic regulation in plants, thereby enhancing the salt tolerance of plants. Betalains are important secondary metabolites. In this paper, our results indicate that salt stress increased the betalain content in amaranth. The contents of betacyanin and betaxanthin were significantly higher than those of the control and other concentrations. They perhaps act as osmoregulatory substances for the plants to help them adapt to salt stress. Betalain production in the leaves of *Portulaca oleracea* increased with saline stress [33].

TCP transcription factors perform comprehensive functions in plant growth and development, such as branching [34,35,36], leaf morphogenesis [37,38,39], and flower development [40,41]. Additionally, the TCP genes are also involved in the response to exogenous factors, such as salt stress and other abiotic stresses [43,44,45,46,47,48,49,50]. The PCF gene, a member of the TCP gene family in *Oryza sativa*, activates the expression of the NHX gene, thereby improving the plant’s salt tolerance [51]. The salt tolerance of the TCP-overexpressing plants was stronger than that of the wild-type plants under salt stress [65]. NHX genes play an important role in plants’ response to salt stress [66]. In order to clarify the function of the *AtrTCP1* gene in the response to salt stress, we transferred pCambia1301-35S-AtrTCP1-GUS into Arabidopsis. We compared the germination rates of the transgenic AtrTCP1 and wild-type (WT) plant seeds under salt stress. The germination rates and timings of the *35S::AtrTCP1*-GUS plants were different from those of the WT plants. The seed germination rate and germination potential of the transformed *35S::AtrTCP1*-GUS plants were higher than those of the wild-type plants, and the former plants germinated earlier under normal conditions. Salt stress delayed seed germination and significantly reduced the germination rate of *Eruca sativa* [67]. However, the germination rate and potential of the transformed plants were significantly higher than those of the wild type. In our study, under the treatment of 150 mmol·L^−1^ NaCl, the germination rate of the WT and *35S::AtrTCP1*-GUS plant seeds was 27.0% on the seventh day and 93.0% on the fourth day, respectively. These results imply that *AtrTCP1* can promote seed germination and enable *Arabidopsis thaliana* seeds to adapt to salt stress.

Under salt stress, the plants underwent morphological, physiological, and metabolic changes [3,4,5,6,7,8]. We found that the transgenic AtrTCP1 and WT *Arabidopsis thaliana* plants were shorter under salt stress than they were under normal conditions, and they had fewer leaves. Meanwhile, the contents of chlorophyll a, chlorophyll b, and total chlorophyll in the salt-stress-induced plants were lower than those in the non-salt-stress-induced plants because it decreased the leaf chlorophyll content [9]. However, the chlorophyll content of the transformed *35S::AtrTCP1*-GUS plants was higher than that of the wild type under salt stress and not under salt stress. *AtrTCP1* perhaps improved the salt tolerance of the *Arabidopsis thaliana* plants. TCP family proteins perform multiple functions in the regulation of flowering. Other studies have shown that that PIP1;1, a member of TCP, can promote bolting and flowering in *Arabidopsis* [68,69]. The transformed *35S::AtrTCP*1 plants bloomed, and the WT grew about 16 leaves after 13 days of treatment. The TCP4 transcription factor plays an important role in plant growth and development, especially in flower development [70]. Under salt stress, the transformed *35S::AtrTCP1* plants bloomed when they grew 21.8 leaves after 16.2 days of treatment. In contrast, the wild type flowered when it grew 21.6 leaves after 18.4 days of treatment. This implies that salt stress delayed the flowering of *Arabidopsis*, but *AtrTCP1* promoted blooming. We speculated that the transformed plants flowered early to resist salt stress. The TCP family proteins participate in the flowering pathways by acting at different levels and by modulating the expression of both flowering time-related and floral meristem identity genes [71]. The interaction of AtTCP4 with the flowering activator GIGANTEA (GI) enhances its DNA-binding ability onto the CO promoter, facilitating CO transcription [72]. Although TCP family proteins have multiple functions in flowering regulation, the molecular basis of their functional specificity is virtually unknown and requires further investigation. The regulation of the expression and activity of *TCP* by environmental conditions could converge to adjust flowering time in response to growth conditions, ensuring reproductive success [72]. In summary, the role of TCP in flowering deserves further investigation.

## 4. Materials and Methods

### 4.1. Experimental Design

The seeds of ‘Suxian No. 1’, provided by Suzhou Academy of Agricultural Sciences, were sowed in plastic pots (10 × 10 cm^2^) filled with nutrient soil [peat moss: perlite, 2:1 (*v*/*v*)] for seed gemination and growth. Four NaCl concentrations, which were 0, 50 mmol·L^−1^, 100 mmol·L^−1^, and 200 mmol·L^−1^, respectively, were added to irrigation water to treat the 3-4-leaf-stage amaranth seedlings. We applied 100 mL of solution every time, with an interval of 3 days between each irrigation, three times in total. The phenotypic characteristics of amaranth plants were observed 3, 6, and 9 days after the last irrigation. Meanwhile, samples were taken for the determination of root activity, pigment content, and antioxidant enzyme activity, and quantitative polymerase chain reaction (qPCR) analysis. These plant seedlings were placed in an illumination incubator at 25 °C, with 60% relative humidity, a 16 h light/8 h dark photoperiod, and 100 μmol m^−2^s^−1^ light intensity.

### 4.2. Measurements and Calculations

#### 4.2.1. Determination of Plant Phenotypes

The plants’ height was measured with a ruler. The washed roots were visualized on a scanner Epson Expression 10000X (Epson, Suzhou, China), and then the total root length (cm) and root volume (the total amount of space occupied by the root, cm^3^) were determined with WinRHIZO software (Pro version 2016a; Regent Instrument Inc., Quebec, QC, Canada).

#### 4.2.2. Root Vitality Determination

The root vitality of the amaranth plants was measured using the triphenyltetrazolium chloride (TTC) method [73]. We prepared 0, 0.0025%, 0.05%, 0.01%, 0.015%, and 0.02% TTC solutions and poured 10 mL of each into test tubes, and then added 10 mL of ethyl acetate and a small amount of Na_2_S2O_4_ (approximately 2.0 mg, with the same quantity in each tube). They were then shaken sufficiently to produce red TTF, with ethyl acetate as the blank reference, to determine the OD of the solutions with a spectrophotometer (485 nm), and then a standard curve was drawn. The TTC standard curve was y = 0.8895x − 0.078 (R^2^ = 0.9943).

A total of 0.2 g amaranth root samples treated with different concentrations of NaCl were extracted and poured into a 10 mL beaker with 0.4% TTC and 66 mmol/L phosphate-buffered solution (pH = 7.0), and then kept at 37 °C for 2 h in the dark. Then, 2 mL of 1 moL·L^−1^ sulfuric acid was added to terminate the reaction. The root was removed, carefully wiped with filter paper, and then ground with 3 mL of ethyl acetate and a small amount of quartz sand in a mortar to extract TTF. A small amount of ethyl acetate was used to wash the residue 2~3 times, and then poured into the test tube. Finally, 10 mL of ethyl acetate was added, and a spectrophotometer was used for colorimetry, taking the blank test (with sulfuric acid, and then the root samples were added) as the reference; the readout was 485 nm OD. According to the standard curve of y = 0.8895x − 0.078(R^2^ = 0.9943), the small amount of TTC can be obtained. Because there is less TTC, it will also become weaker.

Reducing Strength of TTC (mg/g/h) = Reduction Amount of TTC (mg)/Weight of Root Sample (g)/Time (h)

#### 4.2.3. Determination of Pigment Contents

The betalain, flavonoid, carotenoid, and chlorophyll contents in the amaranth plants were determined. The betalains in the *Amaranthus tricolor* leaves were extracted according to Liu. The betacyanins and betaxanthins were detected spectrophotometrically at the 538 and 470 nm wavelengths and quantified using the molar extinction coefficients 60,000 and 48,000 M^−1^cm^−1^, respectively. The flavonoid content in *Amaranthus tricolor* was determined according to a flavonoid extraction and determination protocol (Comin Biotechnology Co., Ltd., Suzhou, China). The chlorophyll and carotenoid contents were analyzed according to Liu [74].

#### 4.2.4. Determination of Leaf Antioxidant Enzyme Activity and MDA Content

According to the determination protocol (Comin Biotechnology Co., Ltd., Suzhou, China), the peroxidase (POD, EC1.11.1.7) and catalase (CAT, EC1.11.1.6) activity levels and MDA content were determined, respectively. We measured the OD using a spectrophotometer at the 470 nm wavelength and read the OD value every minute. The OD value changed, indicating POD enzyme activity, which is represented by (A_test_470 − A_blank_470)/[min·FW (g)]. We measured the OD at the ultraviolet 240 nm wavelength and read the OD value every minute. The OD value changed, indicating CAT enzyme activity, which is represented by (A_test_240 − A_blank_240)/[min·FW (g)]. We measured the OD using a spectrophotometer at the 532 nm and 600 nm wavelengths. The MDA content is represented by 53.763 × [(A532_test_ − A532_blank_) − (A600_test_ − A600_blank_)]/FW (g). The MDA content is represented by 53.763 × [(A532_test_ − A532_blank_) − (A600_test_ − A600_blank_)]/FW (g).

#### 4.2.5. Transformation in Arabidopsis and Salt Treatment

Our previous study showed that the *AtrTCP*1 gene is expressed under salt stress using qRT-PCR analysis [53]. However, its function is still unclear. Thus, the full-length open reading frame (ORF) of AtrTCP1 was cloned using the amplification system (94 ° C for 3 min, followed by 35 cycles of 94 °C for 30 s, 55 °C for 30 s, 72 °C for 45 s, and 72 °C for 7 min). Then, PCR was conducted on the plants that overexpressed vector pCambia1301 [carrying a glucuronidase (GUS) tag], and then transformed into the *Agrobacterium tumefaciens* strain GV3101 (the primers are listed in Supplementary Table S1).

The bacterial cells containing the *35S:AtrTCP1-GUS* vector were cultivated in liquid Luria–Bertani (LB) medium with 50 μg/mL kanamycin (Kan) and 50 μg/mL rifampicin (Rif) in a constant temperature shaker at 200 rpm at 28 °C overnight. Subsequently, the bacterium cells were collected via centrifugation at 6000 rpm at 28 °C for 5 min. The OD_600_ of GV3101 bacterial liquid was adjusted to 0.9–1.1 with freshly prepared 5% (*w*/*v*) sucrose solution and 0.02% Silwet L-77.

The *Arabidopsis* seeds were sown on in plastic pots (10 × 10 cm^2^) filled with nutrient soil [peat moss: perlite, 2:1 (*v*/*v*)] for seed gemination. We grew healthy seedlings until they developed floral inflorescences. We transformed the empty vector (pCambia1301: empty) and *35S:AtrTCP1-GUS* into *Arabidopsis*, respectively, by using the floral dip method. The T0 generation Arabidopsis seeds were planted in the MS medium containing 25 g/mL hygromycin (Hyg) to screen the positive seedlings. The positive seedlings with 2–4 green leaves were transplanted to nutrient soil [peat moss: perlite, 2:1 (*v*/*v*)]. After about one month, we strained the leaves of the T1 generation transgenic plants and WT with glucuronidase solution. Meanwhile, we extracted RNA to further verify the positive seedlings with PCR using the *AtrTCP1*, *GUS,* and *Hyg* primer genes (Supplementary Table S1). The transgenic *Arabidopsis* plants were bred and screened until T2 generation lines were obtained. Because the *AtrTCP1* gene in amaranth does not exist in *Arabidopsis*, and the relationship between *AtrTCP1* in amaranth and *AtTCP20* in *Arabidopsis* is relatively close [53], we designed a pair of primers for simultaneously detecting the *AtrTCP1* expressional level according to the *AtTCP20* and *AtrTCP1* gene sequences. The transcription of *AtTCP20* (*AtrTCP1*) in the T2 generation transgenic *Arabidopsis* plants was examined using RT-qPCR (Supplementary Table S1).

The pure T2 generation lines and wild-type *Arabidopsis* seeds were sown in Petri dishes with three layers of filter paper, and then 50 mL of either 150 mmol·L^−1^NaCl solution or in distilled water was added for gemination. We determined the seed germination potential and germination rate on the 3rd and 7th days, respectively. The number of germination days represents the number of days from sowing to germination.

Meanwhile, we sowed the same seeds in plastic pots (10 × 10 cm^2^) filled with nutrient soil [peat moss: perlite, 2:1 (*v*/*v*)] for seed gemination and plant growth for 15 days. The seedlings with more than 6 leaves were treated with 150 mmol·L^−1^ NaCl solution or distilled water. After 7 days, we recorded the phenotypes of these plantlets and determined the chlorophyll contents. Lastly, the transcription of *AtrTCP1* and *GUS* in the plants was examined using RT-qPCR.

Total RNA was isolated from samples using MolPure Plant Plus RNA Kit (Yeasen, Beijing, China) according to the manufacturer’s instructions. First-strand cDNA was synthesized from 1 mg of total RNA using Recombinant M-MLV reverse transcriptase (TransGen Biotech, Beijing, China). Quantitative real-time PCR (qRT-PCR) was performed in optical 96-well plates using the Roche Light Cycler 480 instrument (Roche, Sweden). The reactions were carried out in a 20 mL volume containing 10 mL of SYBR Premix Ex Taq (Yeasen, Beijing, China), 0.8 mL of gene specific primers, 2 mL of diluted cDNA, and 6.4 mL of ddH_2_O. The PCR conditions were as follows: 94 ° C for 3 min, followed by 35 cycles of 94 °C for 30 s, 58 °C for 15 s, 72 °C for 15 s, and then 72 °C for 7 min), and the 2^−ΔΔCt^ method was used for the quantitative analysis of gene expression [74].

### 4.3. Statistical Analysis

The data are presented as mean ± standard error and were subjected to analysis of variance (ANOVA). The means were compared using the ad hoc Tukey test (*p* < 0.05%). All the statistical analyses were performed using SPSS 20 (IBM Corp., Armonk, NY, USA). GraphPad Prism 6.01 (GraphPad Software Inc., La Jolla, CA, USA) was used to draw bar charts.

## 5. Conclusions

Amaranth shoot and root growth was inhibited by a 200 mmol·L^−1^ NaCl solution, but it was promoted by 50 mmol·L^−1^. Meanwhile, the plants also showed physiological responses, which included salt-induced injuries and adaptation to salt stress. Moreover, the germination day of the *35S::AtrTCP1-GUS Arabidopsis* seeds was earlier than that of the wild type (WT) under normal conditions. Under treatment with 150 mmol·L^−1^ NaCl, the germination rate of the *35S::AtrTCP1-GUS* plant seeds was higher than that of the WT. Meanwhile, the germination day of the *35S::AtrTCP1-GUS Arabidopsis* seeds was also earlier than that of the WT. Under salt stress, the transformed *35S::AtrTCP1* plants bloomed earlier than the WT.

## Figures and Tables

**Table 1:** Effect of salt stress on the height of amaranth plants.

Days after Treatment/d	NaCl Concentration			
	0 mmol·L^−1^	50 mmol·L^−1^	100 mmol·L^−1^	200 mmol·L^−1^
3	8.39 ± 0.33 b	9.33 ± 0.58 a	8.36 ± 0.42 b	6.10 ± 0.41 c
6	8.52 ± 0.14 b	9.60 ± 0.42 a	9.60 ± 0.42 a	6.27 ± 0.28 c
9	8.88 ± 0.26 b	9.87 ± 0.17 a	9.70 ± 0.45 a	6.71 ± 0.28 c

Note: Data are presented as mean ± standard error. The different lowercase letters represent significant differences at the 0.05 level.

## Data Availability

All datasets generated for this study are included in the article.

## Supplementary Materials

The following supporting information can be downloaded at: https://www.mdpi.com/article/10.3390/ijms25105437/s1.
